# Variations and inter-relationship in outcome from emergency admissions in England: a retrospective analysis of Hospital Episode Statistics from 2005–2010

**DOI:** 10.1186/1472-6963-14-270

**Published:** 2014-06-19

**Authors:** Peter James Edward Holt, Sidhartha Sinha, Baris Ata Ozdemir, Alan Karthikesalingam, Jan Dominik Poloniecki, Matt Merfyn Thompson

**Affiliations:** 1 Department of Outcomes Research, St George’s University of London, London, UK; 2 St George’s Vascular Institute, St George’s Hospital, Blackshaw Road, Tooting, London SW17 0QT, UK

**Keywords:** Quality of Health Care [MeSH], Benchmarking [MeSH], Outcomes assessment (Health Care) [MeSH], Health services research [MeSH], Mortality [MeSH], General surgery [MeSH], Orthopaedics [MeSH], Myocardial infarction [MeSH], Stroke [MeSH], Sepsis [MeSH], Femoral neck fractures [MeSH]

## Abstract

**Background:**

The quality of care delivered and clinical outcomes of care are of paramount importance. Wide variations in the outcome of emergency care have been suggested, but the scale of variation, and the way in which outcomes are inter-related are poorly defined and are critical to understand how best to improve services. This study quantifies the scale of variation in three outcomes for a contemporary cohort of patients undergoing emergency medical and surgical admissions. The way in which the outcomes of different diagnoses relate to each other is investigated.

**Methods:**

A retrospective study using the English Hospital Episode Statistics 2005–2010 with one-year follow-up for all patients with one of 20 of the commonest and highest-risk emergency medical or surgical conditions. The primary outcome was in-hospital all-cause risk-standardised mortality rate (in-RSMR). Secondary outcomes were 1-year all-cause risk-standardised mortality rate (1 yr-RSMR) and 28-day all-cause emergency readmission rate (RSRR).

**Results:**

2,406,709 adult patients underwent emergency medical or surgical admissions in the groups of interest. Clinically and statistically significant variations in outcome were observed between providers for all three outcomes (p < 0.001). For some diagnoses including heart failure, acute myocardial infarction, stroke and fractured neck of femur, more than 20% of hospitals lay above the upper 95% control limit and were statistical outliers. The risk-standardised outcomes within a given hospital for an individual diagnostic group were significantly associated with the aggregated outcome of the other clinical groups.

**Conclusions:**

Hospital-level risk-standardised outcomes for emergency admissions across a range of specialties vary considerably and cross traditional speciality boundaries. This suggests that global institutional infra-structure and processes of care influence outcomes. The implications are far reaching, both in terms of investigating performance at individual hospitals and in understanding how hospitals can learn from the best performers to improve outcomes.

## Background

Wide variations in clinical outcomes between hospitals have been described for a number of conditions. In addition, a number of high-profile failures of healthcare have been reported [1,2]. One common theme has been a failure to deliver safe and high quality care, with subsequent poor clinical outcomes. In response to such variations and failures, there have been suggestions that rating systems for hospitals could be developed in tandem with stringent hospital inspections. The aim of such a system would be to inform key stakeholders on the quality of care delivered by providers and to provide a method to prevent problems from developing, detect failure before harm is done and to facilitate a timely response.

The feasibility of a single rating being able to achieve all these aims has been questioned by commentators, including a report by the Nuffield Trust [3,4]. In particular, a rating system can only be useful if significant variations in outcome exist between hospitals, and can only be of value if the outcomes of different diagnoses are inter-related within individual hospitals [5]. Whether ratings should be limited to specific conditions, or whether they can be hospital-wide requires evaluation [6]. Therefore, it is important to define whether hospitals perform at a similar level of outcome across a range of conditions, or whether individual providers encompass a range of performance levels, dependent on specialty [7-9].

The objectives of this study were to determine whether the risk-standardised clinical outcomes for emergency admissions varied significantly between providers, and whether outcomes were inter-related between different diagnoses at a hospital level.

## Methods

The data source for this retrospective study was the national administrative dataset, the Hospital Episode Statistics (HES) data, for the period 1^st^ April 2005 to 31^st^ March 2010. The HES detail every admission into the NHS and allow patients to be tracked between NHS hospitals and across years through the use of a unique pseudo-anonymised identifier. The dataset can be considered an inclusive record of National Health Service (NHS) hospital activity in England as there is a requirement for every hospital to submit a “minimum dataset” to the Department of Health. Each hospital submits data from its own Patient Administration System (PAS) to the centralised HES data warehouse which is then validated and cleaned by the NHS Information Centre according to a pre-specified list of rules. HES contains publicly available admitted patient care data from 1989 onwards, with more than 12 million new records added each year. Each HES record contains a significant amount of information about the associated admission including patient demographics, the treating hospital, diagnostic and procedural coding, length of stay, discharge status and destination. Emergency and elective admissions can be differentiated by specific combinations of codes relating to type of admission and diagnostic/procedural code sub-groups. As a result, it is possible to identify patient cohorts with specific characteristics with a high degree of accuracy [10]. A recent report based on HES data describes the approximate number of emergency admissions in England as more than 4.5 million per annum [11].

A five-year study period was chosen to achieve a pragmatic balance between sample size and the cohort reflecting contemporary practice. The HES were linked with the Office of National Statistics (ONS) Registry data to provide longer-term and out-of-hospital mortality data. Clinical coding in the HES is through the OPCS-4 procedural coding system and ICD-10 diagnostic codes [12].

Twenty patient groups were used that covered the breadth of hospital emergency admissions in adults (>17years of age) to acute NHS (public) hospitals. The groups were chosen a-priori by quantifying the commonest emergency admission diagnoses with the highest numbers of deaths to all acute NHS hospitals using a sample of HES data (2010/2011) (Additional file 1: Tables S1 and S2). ICD-10, OPCS-4 and HRG codes were used to create clinically meaningful groups. Medical conditions were defined by ICD-10 codes whilst surgical groups were defined by OPCS-4 codes.

The primary outcome measure was in-hospital all-cause risk-standardised mortality rate (in-RSMR) and secondary outcomes were one-year all-cause risk-standardised mortality rate (1yr-RSMR) and 28-day all-cause emergency readmission rate (RSRR) [13]. In-hospital mortality was defined as death occurring after admission to an index hospital and before discharge either from the index hospital or any subsequent receiving hospital in cases where the patient was transferred from the index hospital (a definition equivalent to a “super-spell” whereby a “spell” is HES-specific terminology denoting one continuous admission at a hospital) [12,14]. RSRRs were quantified using only patients discharged alive from hospital.

Established methods were used to extract and clean the data, and entries with missing key fields, such as operation dates, were excluded from analyses (representing 1.15% of the dataset) [15-17]. Each patient was included only once, using the diagnostic or procedural code in the primary field in the first occurrence for allocation to a clinical group. This prevented a single patient being included multiple times due to complications of care, or multiple surgical procedures.

The confounding effect of inter-hospital transfers on in-RSMR and RSRR was accounted for as patients can be tracked between hospitals within the HES. Previous studies have shown that linking concurrent admissions together and assigning the ultimate outcome to the index hospital (super-spells) provides the most accurate reflection of in-hospital death rates [12].

### Statistical analyses

The analyses had two distinct stages: first, to quantify the extent of variations in outcome within each clinical group; and second, to determine whether the outcomes of a specific clinical group were associated with the collective outcomes of the other clinical groups within the same hospital. Statistical analysis was performed using SAS version 9.2 (SAS Institute, USA).

Risk-standardisation was performed using hierarchical logistic regression models. Patient age, sex, RCS (Royal College of Surgeons) Charlson score, social deprivation index and year of admission formed first-level predictors [18]. Co-morbidity scores for cases were derived from the HES data by using published methodology [19]. The method relies on identification of pre-existing ICD-10 diagnostic codes to denote co-morbidity. The score is created by calculating the number of co-morbidity categories present in any admission episode in the preceding 365 days or in the index episode for any one case of interest. A case scoring in any one category (regardless of the number of times) achieves a score of 1, scoring in any two different categories achieves a score of 2 and a score in three or more categories achieves a score of 3. Cases without any flagged co-morbidities receive a score of 0. The scoring system is a modification (the Royal College of Surgeon’s [RCS] modification) of the original score derived by Charlson [20,21]. The Index of Multiple Deprivation (IMD) overall ranking is made by combining seven weighted domains [22]. A score of 1 indicates the most deprived and 32482 the least deprived. This numerical range is sub-divided into fifths and cases are allocated into to one of the five quintiles according to their score (i.e. quintile 1 represents the most deprived cases and quintile 5 represents the least deprived cases).

The second level of the RSMR and RSRR models permitted hospital-level random intercepts to vary in order to identify hospital-specific random effects and account for the clustering of patients within the same hospital [23]. This allowed, within- and between-hospital variation to be separated, within the limits of the data, after adjusting for patient-level characteristics. Analysis was performed at NHS trust level (i.e. potentially including more than one physical site) and the term “hospital” is used synonymously with “trust”.

#### Variation in outcome

Variations in outcome for each metric were assessed for each clinical group. Risk estimates from individual patient data were used to calculate the expected number of deaths for each condition at each hospital. For each clinical group, the discrepancy between the observed and expected mortality in each hospital was quantified. A statistically significant divergence was reported when it exceeded the 95% confidence interval of the Poisson distribution. Visually these were represented with risk-standardised funnel plots.

#### Outcome inter-relationship analysis

The risk-standardised outcome for each of the twenty clinical groups was compared against the risk-standardised aggregate outcome for the other clinical groups excluding the procedure/condition of interest (e.g. acute myocardial infarction vs. an aggregate outcome of the other nineteen groups [i.e. excluding acute myocardial infarction]) using published methodology [8,14].

Hospitals were placed into quintiles containing equal numbers of patients based on their aggregate events rates. This was achieved by ranking hospitals according to their aggregate “other” event rate into five groups (quintiles) such that hospitals with the highest observed event rate relative to expectation (calculated by dividing the difference between the observed and expected numbers by a measure of random variability) were in the highest numbered group (quintile 5) and those with the lowest observed event rate relative to expected were in quintile 1. Hospitals with very high event rates relative to expected would not necessarily be allocated to the highest quintile if the case load was small. Aggregation was performed using the provider’s mean Studentised residual aggregated across the remaining patient groups such that conditions with high event rates did not dominate the aggregation. Hospitals were assigned such that there were roughly equal numbers of patients in each quintile. Having assigned quintiles of aggregate “other” event rates, the combined event rate for the procedure/condition of interest was calculated for each quintile (by multiplying the observed to expected ratio by the crude total death rate for the procedure/condition of interest). Results were expressed as a bar plot. The relationship between the outcome for each clinical group and the amalgamated outcome quintiles was tested using logistic regression.

Reporting of the study complied with the STROBE guidelines [24].

### Ethics committee approval

The Wandsworth research ethics committee (WanREC) confirmed that no ethical approval was required for this study.

## Results

2,406,709 admissions were included across 20 emergency groups in the five-year period. Summary demographic and outcome data for each group are provided (Table 1).

**Table 1:** Demographic data and crude mortality outcomes for emergency medical diagnoses and emergency surgical procedures

**Diagnosis/procedure Abbreviation**	**Acute myocardial infarction AMI**	**Acute heart failure CCF**	**Stroke CVA**	**Pneumonia LRTI**	**Pulmonary embolism PE**	**Urosepsis UTI**	**Sepsis SEPSIS**	**Cardiac arrest ARREST**	**Acute pancreatitis PANC**	**Fractured neck of femur NOF**
*Number of cases/procedures*	296673	206400	289806	458064	74282	390616	110240	13168	58159	185567
*Mean patient age (years [SE])*	70.2 (0.026)	78.4 (0.025)	75.2 (0.025)	70.9 (0.027)	64.4 (0.063)	69.5 (0.035)	65.7 (0.061)	70.0 (0.138)	56.1 (0.080)	80.9 (0.026)
*Male proportion (% [SE])*	63.2 (0.0009)	50.0 (0.0011)	47.4 (0.0009)	50.8 (0.0007)	47.0 (0.0018)	34.3 (0.0008)	49.3 (0.0015)	59.3 (0.0043)	52.3 (0.0021)	25.1 (0.0010)
*RCS Charlson co-morbidity score (%)*	0(49.2)	0(0)	0(0)	0(32.3)	0(0)	0(45.0)	0(32.1)	0(37.3)	0(64.7)	0(46.5)
	1(29.6)	1(32.4)	1(51.2)	1(36.2)	1(58.9)	1(31.2)	1(35.6)	1(32.2)	1(25.1)	1(34.2)
	2(13.3)	2(35.4)	2(31.9)	2(19.5)	2(28.3)	2(15.1)	2(19.9)	2(18.2)	2(7.4)	2(13.4)
	3(7.8)	3(32.2)	3(16.8)	3(12.0)	3(12.8)	3(8.7)	3(12.4)	3(12.3)	3(2.8)	3(5.9)
*Social deprivation quintile (%)*	1(21.4)	1(22.9)	1(20.7)	1(25.1)	1(19.5)	1(24.1)	1(22.8)	1(21.9)	1(27.0)	1(18.5)
	2(20.7)	2(21.4)	2(20.4)	2(21.5)	2(19.7)	2(21.8)	2(21.0)	2(20.6)	2(21.7)	2(19.6)
	3(20.7)	3(20.7)	3(20.7)	3(19.7)	3(20.6)	3(20.0)	3(20.0)	3(20.0)	3(19.1)	3(21.1)
	4(19.8)	4(19.0)	4(20.3)	4(18.1)	4(20.6)	4(18.4)	4(19.1)	4(19.4)	4(17.3)	4(21.3)
	5(17.5)	5(16.1)	5(18.1)	5(15.6)	5(19.6)	5(15.8)	5(17.1)	5(18.1)	5(15.1)	5(19.5)
*In-hospital mortality rate (% [SE])*	12.1 (0.0006)	17.0 (0.0008)	24.8 (0.0008)	26.3 (0.0007)	8.0 (0.0010)	5.1 (0.0004)	27.7 (0.0013)	66.7 (0.0041)	5.7 (0.0010)	9.8 (0.0007)
*1-year mortality rate (% [SE])*	22.8 (0.0008)	41.9 (0.0011)	37.5 (0.0009)	42.9 (0.0007)	21.8 (0.0015)	24.3 (0.0007)	44.6 (0.0015)	72.1 (0.0039)	10.3 (0.0013)	28.4 (0.0010)
*28-day emergency readmission rate (% [SE])*	28.6 (0.0008)	36.9 (0.0011)	19.0 (0.0007)	29.2 (0.0007)	24.2 (0.0015)	29.4 (0.0007)	33.2 (0.0014)	26.4 (0.0038)	25.1 (0.0018)	20.0 (0.0009)
*Median (IQR) length of stay (days)*	6 (7)	7 (11)	11 (24)	6 (9)	6 (6)	4 (9)	6 (11)	14 (21)	6 (7)	16 (18)
**Diagnosis/procedure**	**Hernia repair**	**Appendicectomy**	**Upper gastrointestinal emergency laparotomy**	**Colorectal emergency surgery**	**Emergency urological surgery**	**Ruptured abdominal aortic aneurysm**	**Endovascular repair of ruptured AAA**	**Emergency carotid endarterectomy**	**Lower limb arterial bypass**	**Lower limb amputation**
**Abbreviation**	**HERNIA**	**APPEND**	**PEPTIC**	**COLOLAP**	**UROL**	**AAA**	**EVAR**	**CEA**	**LEAB**	**AMP**
*Number of procedures*	42302	141538	10237	42366	51963	6687	729	1234	10267	16411
*Mean patient age (years [SE])*	63.2 (0.091)	33.9 (0.042)	59.4 (0.195)	65.5 (0.081)	59.0 (0.083)	73.5 (0.102)	73.5 (0.410)	71.0 (0.280)	70.3 (0.141)	66.5 (0.125)
*Male proportion (% [SE])*	57.0 (0.0024)	51.7 (0.0013)	58.5 (0.0049)	47.5 (0.0024)	67.0 (0.0021)	83.3 (0.0046)	79.4 (0.0150)	66.5 (0.0134)	62.9 (0.0048)	70.2 (0.0036)
*RCS Charlson co-morbidity score (%)*	0(65.3)	0(88.7)	0(65.7)	0(35.3)	0(43.4)	0(0.5)	0(3.2)	0(0.65)	0(14.1)	0(12.6)
	1(24.3)	1(10.3)	1(23.5)	1(42.4)	1(33.9)	1(59.7)	1(44.2)	1(49.2)	1(40.0)	1(27.4)
	2(7.5)	2(0.9)	2(7.9)	2(16.1)	2(15.6)	2(28.2)	2(31.1)	2(31.0)	2(28.1)	2(32.4)
	3(3.0)	3(0.2)	3(2.9)	3(6.3)	3(7.2)	3(11.6)	3(21.5)	3(19.2)	3(17.8)	3(27.6)
*Social deprivation quintile (%)*	1(21.2)	1(20.8)	1(31.3)	1(19.3)	1(20.6)	1(17.6)	1(19.4)	1(18.0)	1(25.8)	1(25.6)
	2(20.9)	2(20.7)	2(21.7)	2(19.7)	2(20.1)	2(20.1)	2(20.4)	2(20.7)	2(21.4)	2(22.3)
	3(20.8)	3(20.0)	3(17.6)	3(20.6)	3(20.2)	3(22.0)	3(19.3)	3(21.0)	3(20.4)	3(20.3)
	4(19.7)	4(19.4)	4(16.2)	4(20.7)	4(19.7)	4(21.6)	4(22.5)	4(22.0)	4(17.4)	4(17.6)
	5(17.4)	5(19.2)	5(13.2)	5(19.8)	5(19.5)	5(18.6)	5(18.5)	5(18.4)	5(15.1)	5(14.2)
*In-hospital mortality rate (% [SE])*	3.1 (0.0008)	0.2 (0.0001)	16.7 (0.0037)	15.7 (0.0018)	3.6 (0.0008)	33.5 (0.0058)	13.2 (0.0125)	1.4 (0.0033)	12.1 (0.0032)	10.5 (0.0024)
*1-year mortality rate (% [SE])*	8.7 (0.0014)	0.5 (0.0002)	22.1 (0.0041)	29.6 (0.0022)	20.7 (0.0018)	38.4 (0.0059)	22.8 (0.0155)	6.8 (0.0072)	26.5 (0.0044)	25.8 (0.0034)
*28-day emergency readmission rate (% [SE])*	15.7 (0.0018)	10.8 (0.0008)	16.3 (0.0037)	23.1 (0.0020)	29.4 (0.0020)	22.3 (0.0051)	31.3 (0.0172)	14.2 (0.0010)	32.8 (0.0046)	25.8 (0.0034)
*Median (IQR) length of stay (days)*	3 (4)	3 (2)	9 (11)	15 (15)	5 (8)	16 (17)	10 (11)	5 (6)	14 (18)	20 (31)

SE = Standard Error.

### Variations in outcomes

Clinically important, and statistically significant, variations in outcome were seen across the range of medical and surgical conditions for in-RSMR, 1 yr-RSMR and RSRR (Table 2).

**Table 2:** Proportions of hospitals with risk-adjusted outcomes for each of the conditions/procedures lying above or below the 95% control limits

**Clinical group**	**In-hospital mortality**		**One-year mortality**		**28-day emergency readmission**	
	**Proportion of hospitals below lower 95% control limit**	**Proportion of hospitals above upper 95% control limit**	**Proportion of hospitals below lower 95% control limit**	**Proportion of hospitals above upper 95% control limit**	**Proportion of hospitals below lower 95% control limit**	**Proportion of hospitals above upper 95% control limit**
*AMI*	16.3%	21.7%	10.2%	17.5%	7.9%	12.1%
*CCF*	17.8%	20.7%	9.8%	16.1%	4.1%	9.3%
*CVA*	19.9%	22.7%	12.2%	19.3%	10.3%	15.4%
*LRTI*	22.2%	28.1%	16.8%	25.4%	7.8%	14.5%
*PE*	10.8%	14.5%	3.0%	6.6%	3.6%	6.0%
*UTI*	14.1%	17.7%	9.4%	16.1%	12.0%	18.2%
*SEPSIS*	17.7%	23.2%	9.9%	10.5%	3.9%	6.2%
*ARREST*	1.8%	2.4%	1.2%	1.2%	0%	5.6%
*PANC*	1.2%	4.9%	3.7%	4.3%	4.3%	1.8%
*NOF*	18.1%	19.4%	8.1%	14.4%	11.3%	14.4%
*HERNIA*	2.5%	6.7%	1.8%	3.7%	1.2%	3.1%
*APPEND*	0.6%	3.1%	1.3%	5.0%	12.6%	15.1%
*PEPTIC*	1.9%	1.2%	0.6%	2.5%	0.6%	4.4%
*COLOLAP*	5.5%	10.4%	1.2%	4.3%	2.4%	3.7%
*UROL*	3.0%	9.5%	4.7%	7.1%	4.7%	7.7%
*AAA*	5.8%	7.2%	4.3%	5.0%	1.5%	4.5%
*EVAR*	0%	2.4%	0%	2.4%	0%	2.5%
*CEA*	0%	0%	0%	3.8%	0%	6.6%
*LEAB*	0%	2.8%	0%	4.2%	0.7%	3.6%
*AMP*	2.4%	8.5%	1.2%	4.2%	0.6%	4.2%

Outcomes shown are risk-standardised in-hospital mortality, one-year mortality and 28-day emergency readmission rates.

#### In-hospital all-cause risk-standardised mortality rate (in-RSMR)

**Figure 1:**
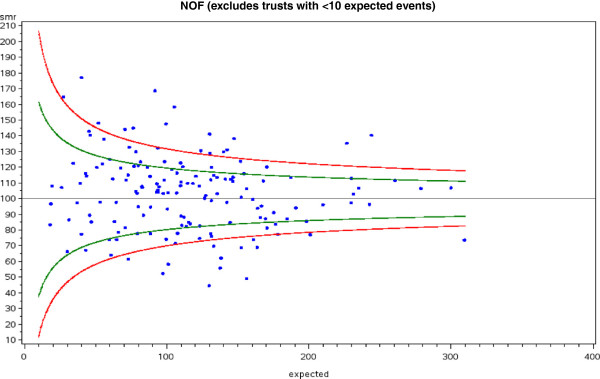
**Example of a funnel plot visually exploring the issue of variability in all-cause in-hospital mortality rate for fractured neck of femur (NOF) between providers.** 37.5% of hospitals lay outside the 95% (green lines) confidence intervals (green) and 18.1% of hospitals lay outside the 99.8% (red lines) confidence intervals. Further statistical analysis determined whether the probability that the number of hospitals outside the confidence intervals was significant (both p < 0.0001).

For in-RSMR, the clinical groups with the greatest proportion of hospitals lying outside statistical control limits were medical diagnoses, including acute myocardial infarction, heart failure, stroke, pneumonia and sepsis. For each of these, more than 20% of hospitals lay above the 95% confidence limit. This was in addition to wide variations in the actual in-RSMR for each diagnosis, with many diagnoses demonstrating more than doubling of mortality rates between hospitals. An illustrative funnel plot for fractured neck of femur is given in Figure 1.

Within surgical diagnoses, significant variations in mortality were seen for fractured neck of femur, emergency colorectal laparotomy, ruptured abdominal aortic aneurysm repair, and emergency urological interventions. Of particular note, for fractured neck of femur, 19.4% of hospitals lay above the upper 95% confidence limit for in-RSMR.

#### One-year all-cause risk-standardised mortality rate (1 yr-RSMR)

**Figure 2:**
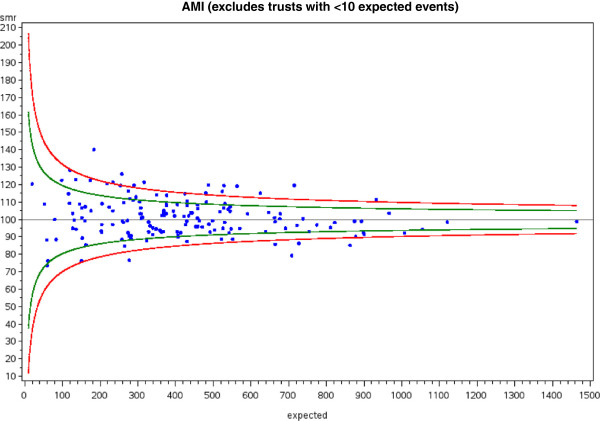
**Example of a funnel plot visually exploring the issue of variability in all-cause 1-year mortality rate for acute myocardial infarction (AMI) between providers.** 27.7% of hospitals lay outside the 95% (green lines) confidence intervals (green) and 11.4% of hospitals lay outside the 99.8% (red lines) confidence intervals. Further statistical analysis determined whether the probability that the number of hospitals outside the confidence intervals was significant (both p < 0.0001).

A number of medical conditions displayed wide variations in 1 yr-RSMR. For acute myocardial infarction, heart failure, stroke, pneumonia and urinary tract infection more than 15% of hospitals lay above the upper 95% confidence limit. An illustrative funnel plot for acute myocardial infarction is given in Figure 2.

Mortality variations after emergency surgery were less pronounced for 1 yr-RSMR than for in-RSMR. Significant variations were still observed, most notably, for fractured neck of femur where 14.4% of hospitals lay above the upper 95% confidence limit for 1 yr-RSMR.

#### 28-day all-cause risk-standardised emergency readmission rate (RSRR)

**Figure 3:**
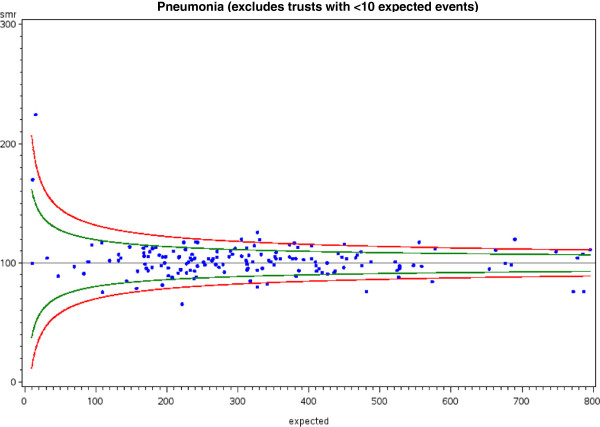
**Example of a funnel plot visually exploring the issue of variability in all-cause 28-day emergency readmission rate for pneumonia (LRTI) between providers.** 22.3% of hospitals lay outside the 95% (green lines) confidence intervals (green) and 8.9% of hospitals lay outside the 99.8% (red lines) confidence intervals. Further statistical analysis determined whether the probability that the number of hospitals outside the confidence intervals was significant (both p < 0.0001).

The widest variations in RSRR were for appendicectomy, fractured neck of femur, urinary tract infections, pneumonia, stroke and acute myocardial infarction with over 10% of hospitals lying above the upper 95% confidence limit. An illustrative funnel plot for pneumonia is given in Figure 3.

### Within provider inter-relationship in outcome between clinical groups

First, significant differences were observed between aggregated outcome quintiles when considering all 20 groups together for in-RSMR, 1 yr-RSMR and RSRR (p < 0.0001 for each). This confirmed hospital-wide variations in the overall outcomes delivered by individual hospitals for acute medical and surgical admissions.

**Figure 4:**
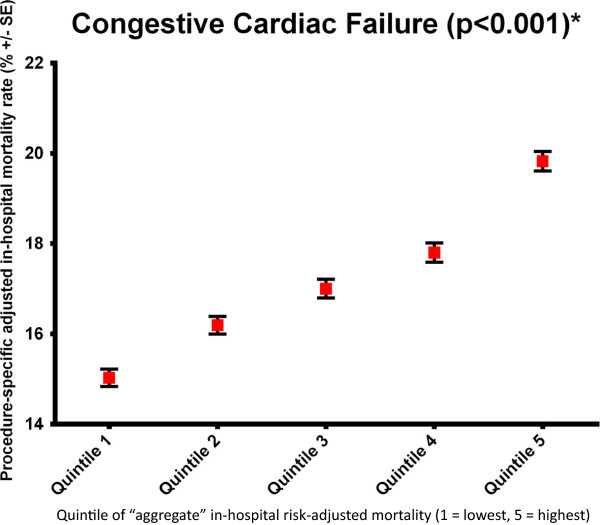
Example of the inter-relationship analysis for heart failure (all-cause in-hospital mortality rate).

**Figure 5:**
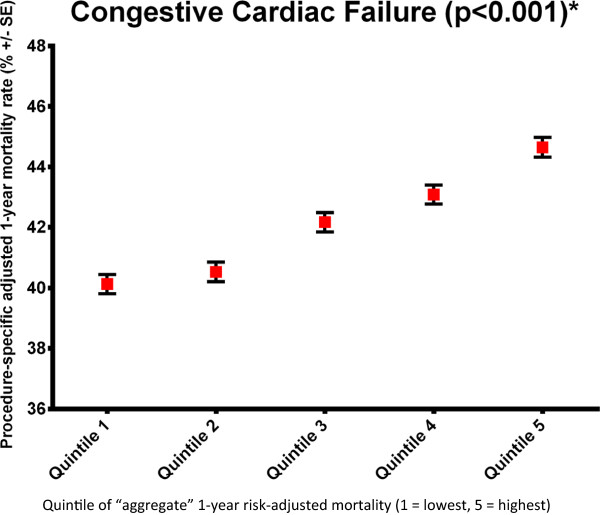
Example of the inter-relationship analysis for heart failure (all-cause 1-year mortality rate).

Second, the outcomes for specific patient groups within a hospital were strongly associated with the aggregate outcome of the other clinical groups within the same hospital (Additional file 1: Tables S3–S5 and Figures S1–S3). For those hospitals with the lowest aggregate in-RSMR (quintile 1), the in-RSMR in most cases was also lowest for each clinical group being tested. Using a more specific example, for acute heart failure, hospitals in aggregate quintile 1 had a in-RSMR of 15.0% whereas hospitals in aggregate quintile 5 had a in-RSMR of 19.8% (Figure 4). The mortality rate increased sequentially from quintile 1 to 5 demonstrating that there was a significant relationship between the aggregate in-RSMR and the heart-failure specific outcome (p < 0.001). This result was maintained for 1 yr-RSMR with 40.1% mortality in quintile 1 and 44.6% in quintile 5 and a progressive increase between quintiles (p < 0.001; Figure 5).

This was also seen in surgical procedures, such as fractured neck of femur in which quintile 1 had an in-RSMR of 7.85% and 11.3% in quintile 5, a 43.9% increase in risk across quintiles. The aggregate outcome was strongly associated with fractured neck of femur-specific outcome (p < 0.001). At one-year, the 1 yr-RSMR was 27.0% for quintile 1 and increased across quintiles to 31.0% for quintile 5 (p < 0.001).

Diagnoses such as sepsis showed similar results with the best outcomes being in quintile 1 for in-RSMR 24.3% rising to 32.4% in quintile 5. This 33.3% risk increase was incremental across quintiles, and demonstrated a strong association with the sepsis-specific outcome (p < 0.001). Similarly, the 1 yr-RSMR for sepsis was 41.1% for quintile 1 rising to 48.7% for quintile 5 (p < 0.001).

**Figure 6:**
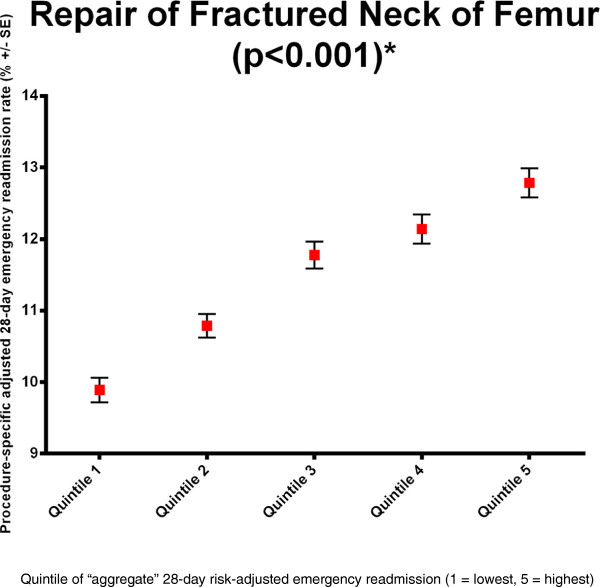
Example of the inter-relationship analysis for fractured neck of femur (all-cause 28-day emergency readmission).

Even for surgical procedures with low event rates the relationships were notable. For example, a doubling of in-RSMR was observed for appendicectomy between quintile 1 (0.119%) and quintile 5 (0.252%), but the aggregate outcome was associated with the appendicectomy-specific outcome for in-RSMR (p = 0.02) and 1 yr-RSMR (quintile 1 = 0.387%, quintile 5 = 0.574%; p = 0.001).When considering RSRR, the aggregated readmission rate was associated with the diagnosis-specific readmission rates in many cases. For example, the RSRR after fractured neck of femur was 9.80% in quintile 1 rising to 13.0% for quintile 5 (Figure 6; p < 0.001).

## Discussion

This study analysed nearly 2.5 million emergency admissions to acute English NHS Trusts over a contemporary five-year period, with one year follow-up. The risk-standardised analyses covered the commonest acute medical and acute surgical diagnoses and procedures using granular, and clinically meaningful subgroups.

The key finding of this study was to demonstrate significant variations between hospitals in terms of the in-hospital mortality rates, one-year mortality rates and 28-day emergency readmission rates that followed non-elective medical or surgical admissions. Furthermore, highly relevant within-provider inter-relationships were seen such that the outcome of a particular condition in one hospital was strongly associated with the aggregated outcome of the other conditions in that hospital.

These data are suggestive of the presence of hospital-level factors that determine the outcomes of a variety of disparate emergency conditions [25,26]. In some cases, this translates into the outcomes from the same diagnosis being significantly better in one hospital than in another hospital. Importantly, in addition to this inter-hospital variation, the outcomes of apparently disparate diagnoses within individual providers are inter-related, such that systemic structural and process factors are implicated that underlie clinical outcomes. These factors remain incompletely defined, but appear to contribute to a system-wide phenomenon that persisted in analyses of 28-day emergency readmission rates and one-year mortality. Whilst it is likely that some of these influences are definable structure or process factors, they might also be representative of more abstract concepts such as institutional attitudes that stimulate excellence [27,28]. The scale of variation between hospital outcomes was concerning, and often exceeded both statistical and clinical boundaries of acceptability. At a time when the quality of care delivered is being closely examined, such variations require further investigation. Modifiable technical, organisational or hospital-related factors play an important role in patient care, and merit further study in order to optimise service delivery and to improve care. Hospital-level factors which have thus far been demonstrated to influence outcomes for certain defined clinical groups include availability of high-dependency beds with adequate medical staffing levels, co-localisation of ancillary specialties, nurse-to-patient ratios, teaching status and hospital size [29-33]. Process factors are more heterogeneous and consequently can be more difficult to identify but have, nonetheless, also been shown to influence outcomes [34-36]. This supposition is further supported by the findings of others that the association between hospital procedural volume and post-operative mortality lacks specificity [9]. Good governance practices such as complete and accurate data collection/submission, internal audit, transparent publication of results and benchmarking against defined quality standards perpetuate good performance. Gauging improvement against national Quality Improvement Frameworks requires objective appraisal of outcomes and must be accompanied by a willingness to change sub-standard practices and to embrace service remodelling if necessary. Robust local monitoring and mandatory submission of data is needed to ensure early reaction to divergent performance and will be of interest to commissioners who may use evidence of such practice to shape service delivery [37]. Transparent reporting of results with clear elaboration of statistical methodology used to risk-adjust data and identify outliers and peer-review of outcomes in an environment seeking to improve quality of care rather than to ostracize individuals or units is essential [14]. Interestingly, current prevailing opinion is that supposed patient empowerment through reporting of clinical outcomes in their current form is unlikely to drive service improvement through market forces as patients tend rely more on subjective information or patient experience measures (PEMs) such as hospital cleanliness [38].

On the basis of these results, the development of an outcomes rating system could be cautiously supported, as this work has shown that there is a need for improved outcomes and reduced variations in outcome in emergency care [39-41]. However, in the first instance, any composite rating system must be based on clinically meaningful subgroups, with definable endpoints. A single pan-provider rating is unlikely to be the correct model [42-44]. These results suggest that acute conditions require detailed appraisal at the speciality level at least. Furthermore, a single rating system that purports to cover both hospitals’ clinical and non-clinical performance will likely be misleading [3].

### Strengths and limitations

Clinical outcomes in this study were limited to validated hard endpoints. As a contemporary five-year cohort was used, concerns about the quality of coding in administrative data are mitigated as systematic reviews have found acceptable coding accuracy rates within the HES data, with data quality improving in more recent years [45,46]. Uniquely, HES data allows for linkage to out-of-hospital death – a powerful feature which adds robustness to the findings through the use of 1-year mortality rates. In addition, recognised and published techniques were used to risk-standardise the data, and to determine the presence and strength of inter-relationships [8,17].

Another strength of the study lies in case selection that focussed on a group of commonly encountered medical and surgical pathologies and ensured a plausible link between mortality and quality by including patients with conditions amenable to salvage [47]. Using the example of colorectal cancer as an example – as the COLOLAP group included only patients who underwent surgery – the majority of patients with disseminated disease requiring only palliation would have been excluded. Similarly the AAA group included only patients who underwent surgery and thus would have excluded moribund patients judged too unfit for surgery. This supports findings that restricting the calculation of standardised mortality ratios to include only certain conditions reduces over-dispersion of data and may yield a more useful comparative statistic [48]. This clinically meaningful case selection addresses the criticism of risk-standardised outcomes that there is a poor correlation between quality of care provided and probability of death [42,49]. Until complete and accurate disease-staged outcomes data are available on the same scale as current administrative data, risk-standardised outcomes remain the best measure of clinical outcomes for national studies. Whilst data from clinical databases and registries may seem more attractive than HES data, their utility remains hampered by the lack of mandatory submission from all providers [39,50].

Finally, the use of a super-spell definition of in-hospital mortality and the inclusion of 1-year mortality and 28-day emergency readmissions as secondary outcome measures demonstrated that the findings were consistent and mitigated criticisms of in-hospital mortality as an outcome measure, namely that it can be confounded by institutional, social and financial factors favouring rapid discharge [51,52]. Despite criticisms of in-hospital mortality as an outcome metric, it should be noted that studies indicate that its value for internal benchmarking purposes is comparable to other mortality outcomes whilst others have demonstrated that different mortality metrics are themselves highly correlated at hospital level [7,53]. With particular regard to health outcomes research using HES data, in-hospital mortality remains the most commonly used metric [12].

Limitations of this study include the possibility of inter-hospital coding variability inherent to the use of retrospective administrative data and it is acknowledged that there remains no universal consensus on risk adjustment methodology with the consequence that different models can yield conflicting results [43,47]. Whilst it is not possible to definitively state that the hospital to hospital variations observed are not at least partially a result of inadequate adjustment for case-mix rather than reflecting genuine differences in quality of care, it should be noted that, with regard to HES data, the covariates used for risk adjustment have been shown to produce regression models of comparable discriminatory power to those derived from clinical databases for colorectal and vascular surgery [7,18,54,55]. Additionally, it is acknowledged that geographic variations in primary care and social care facilities are not included in the model and as such there may be unmeasured confounding given the effect that these factors can have on mortality and readmission rates. Interestingly however, none of the standardised funnel plots showed evidence of over-dispersion of data suggesting that the influence of uncontrolled factors in the risk-adjustment process was not significant [14,56].

The RCS Charlson score has been specifically modified and validated against the HES data [19]. Nonetheless, criticism has been directed towards the use of the Charlson co-morbidity index for case mix adjustment as the index is itself a function of coding depth and accuracy and consequently displays non-constant risk relationships amongst hospitals [43]. In the present study, a post-hoc sensitivity analysis performed by excluding the RCS Charlson score as a regression covariate did not alter the present findings (data not shown). This finding is consistent with research suggesting that exclusion of Charlson scores when calculating RSMRs has only a modest effect and that co-morbidity recording in HES data is not associated with widespread bias [48]. The use of the RCS version of the Charlson score in the analyses acknowledges the finding that modifications of the original Charlson co-morbidity index (i.e. that originally described for US administrative data) to take account of local coding practices can improve the discriminatory power of logistic regression models [57].

The use of mortality as a metric for assessing the quality of care has been criticised although it remains a commonly used measure in health outcomes research and is particularly relevant in the context of emergency admissions [12,14,42].

It is acknowledged that the study does not address which critical structural and process measures account for the observed inter-relationships and that the results are not necessarily generalisable to paediatric populations, elective care admissions or to healthcare systems outside the United Kingdom. Determination of the underlying structures and processes of care remains the focus of ongoing research.

## Conclusions

For emergency medical and surgical admissions in England, wide variations in outcome exist between providers. In addition, strong associations in outcome were found between disparate clinical groups within individual providers that suggested the presence of underlying global structure and process factors underpinning clinical outcomes. These results have implications for the way in which care is delivered and provides potential targets for global quality improvement.

## Competing interest

The authors declare that they have no competing interest.

## Authors’ contributions

PJEH: conception, design, analysis, drafting and critical revision. SS: conception, design, analysis, drafting and critical revision. BAO: design and drafting. AK: drafting and critical revision. JDP: design, analysis, drafting and critical revision. MMT: drafting, critical revision and guarantor. All authors read and approved the final manuscript.

## Pre-publication history

The pre-publication history for this paper can be accessed here:

http://www.biomedcentral.com/1472-6963/14/270/prepub

## Supplementary Material

Additional file 1: Table S1 Clinical group descriptors by diagnostic and procedural codes. **Table S2.** Highest numbers of deaths by OPCS-4, ICD-10 and HRG codes providing the basis for the selection of the 20 emergency groups. **Table S3.** Results from the inter-relationship quintile analysis for in-hospital all-cause risk-stratified mortality. **Table S4.** Results from the inter-relationship quintile analysis for 1-year all-cause risk-stratified mortality. **Table S5.** Results from the inter-relationship quintile analysis for all-cause risk-stratified 28-day emergency readmissions. **Figure S1.** Figures from the inter-relationship quintile analysis for in-hospital all-cause risk-stratified mortality. **Figure S2.** Figures from the inter-relationship quintile analysis for 1-year all-cause risk-stratified mortality. **Figure S3.** Figures from the inter-relationship quintile analysis for all-cause risk-stratified 28-day emergency readmissions. Click here for file
