# Supplementary material for: Variations and inter-relationship in outcome from emergency admissions in England: a retrospective analysis of Hospital Episode Statistics from 2005–2010
Source: BMC Health Serv Res. 2014 Jun 19;14:270. doi: 10.1186/1472-6963-14-270 (PMC4099147; doi:10.1186/1472-6963-14-270)
Supplement: Additional file 1: Table S1 — Clinical group descriptors by diagnostic and procedural codes. Table S2. Highest numbers of deaths by OPCS-4, ICD-10 and HRG codes providing the basis for the selection of the 20 emergency groups. Table S3. Results from the inter-relationship quintile analysis for in-hospital all-cause risk-stratified mortality. Table S4. Results from the inter-relationship quintile analysis for 1-year all-cause risk-stratified mortality. Table S5. Results from the inter-relationship quintile analysis for all-cause risk-stratified 28-day emergency readmissions. Figure S1. Figures from the inter-relationship quintile analysis for in-hospital all-cause risk-stratified mortality. Figure S2. Figures from the inter-relationship quintile analysis for 1-year all-cause risk-stratified mortality. Figure S3. Figures from the inter-relationship quintile analysis for all-cause risk-stratified 28-day emergency readmissions. [file 1472-6963-14-270-S1.pdf]

eTables / eFigures

1) eTable 1: Clinical group descriptors by diagnostic and procedural codes.

2) eTable 2: Highest numbers of deaths by OPCS-4, ICD-10 and HRG codes providing the basis for the selection of the 20 emergency groups.

3) eTable 3: Results from the inter-relationship quintile analysis for in-hospital all-cause risk-stratified mortality.

4) eTable 4: Results from the inter-relationship quintile analysis for 1-year all-cause risk-stratified mortality.

5) eTable 5: Results from the inter-relationship quintile analysis for all-cause risk-stratified 28-day emergency readmissions.

6) eFigure 1: Figures from the inter-relationship quintile analysis for in-hospital all-cause risk-stratified mortality.

7) eFigure 2: Figures from the inter-relationship quintile analysis for 1-year all-cause risk-stratified mortality.

8) eFigure 3: Figures from the inter-relationship quintile analysis for all-cause risk-stratified 28-day emergency readmissions.

Emergency medical condition/surgical procedure	ICD-10 codes (medical)/OPCS-4 codes (surgical)	Additional logic
Acute myocardial infarction (AMI)	I21 Acute myocardial infarction<br>I22 Subsequent myocardial infarction	Emergency mode of admission, primary diagnosis only
Congestive cardiac failure (CCF)	I50 Heart failure<br>I110 Hypertensive heart disease with heart failure	Emergency mode of admission, primary diagnosis only
Stroke (CVA)	I61 Intracerebral haemorrhage<br>I63 Cerebral infarction<br>I64 Stroke, not specified as haemorrhage or infarction	Emergency mode of admission, primary diagnosis only
Pneumonia (LRTI)	J10 Influenza due to other identified influenza virus<br>J11 Influenza, virus not identified<br>J12 Viral pneumonia NEC*<br>J13 Pneumonia due to Streptococcus pneumonia<br>J14 Pneumonia due to Haemophilus influenzae<br>J15 Bacterial pneumonia NEC*<br>J16 Pneumonia due to other organisms NEC*<br>J17 Pneumonia in bacterial diseases EC**<br>J18 Pneumonia, organism unspecified	Emergency mode of admission, primary diagnosis only
Pulmonary embolism (PE)	I26 Pulmonary embolism	Emergency mode of admission, primary diagnosis only
Urinary tract infection (UTI)	N390 Urinary tract infection, site not specified<br>N30 Cystitis	Emergency mode of admission, primary diagnosis only
Sepsis (SEPSIS)	A40 Streptococcal sepsis<br>A41 Other sepsis<br>A392-A394 Meningococcaemia<br>R508 Other specified fever<br>R509 Fever, unspecified	Emergency mode of admission, primary diagnosis only

	R572 Septic shock<br>R578 Other shock<br>R579 Shock, unspecified	
Cardiac Arrest (CA)	I46 Cardiac arrest	Emergency mode of admission, primary diagnosis only
Pancreatitis (PANC)	K85 Acute pancreatitis	Emergency mode of admission, primary diagnosis only
Repair of fractured neck of femur (NOF)	W191 Primary open reduction of fracture of neck of femur and open fixation using pin and plate<br>W241 Closed reduction of intracapsular fracture of neck of femur and fixation using nail or screw<br>W461 Primary prosthetic replacement of head of femur using cement<br>W468 Other specified prosthetic replacement of head of femur using cement<br>W469 Unspecified prosthetic replacement of head of femur using cement<br>W471 Primary prosthetic replacement of head of femur not using cement<br>W478 Other specified prosthetic replacement of head of femur not using cement<br>W479 Unspecified prosthetic replacement of head of femur not using cement<br>W481 Primary prosthetic replacement of head of femur NEC*<br>W488 Other specified other prosthetic replacement of head of femur<br>W489 Unspecified other prosthetic replacement of head of femur	Emergency mode of admission, primary procedure only
Emergency hernia surgery (HERNIA)	T97 Repair of recurrent umbilical hernia<br>T20 Repair of primary inguinal hernia<br>T21 Repair of recurrent inguinal hernia<br>T22 Repair of primary femoral hernia<br>T23 Repair of recurrent femoral hernia<br>T24 Repair of umbilical hernia<br>T25 Repair of incisional hernia	Emergency mode of admission, primary procedure only

	T26 Repair of recurrent incisional hernia<br>T27 Repair of ventral hernia	
Emergency appendicectomy (APPEND)	H01 Emergency excision of appendix<br>H028 Other specified other excision of appendix<br>H029 Unspecified other excision of appendix<br>H03 Drainage of abscess of appendix	Emergency mode of admission, primary procedure only
Emergency peptic ulcer surgery (PEPTIC)	G351 Closure of perforated ulcer of stomach<br>G352 Closure of ulcer of stomach NEC<br>G358 Other specified operations on ulcer of stomach<br>G359 Unspecified operations on ulcer of stomach<br>G362 Closure of perforation of stomach NEC<br>G412 Repair of perforation of pylorus<br>G521 Closure of perforated ulcer of duodenum<br>G522 Suture of ulcer of duodenum NEC<br>G523 Oversew of blood vessel of duodenal ulcer<br>G528 Other specified operations on ulcer of duodenum<br>G529 Unspecified operations on ulcer of duodenum<br>G532 Closure of perforation of duodenum NEC	Emergency mode of admission, primary procedure only
Emergency colorectal laparotomy (COLOLAP)	H041 Panproctocolectomy and ileostomy<br>H05 Total colectomy<br>H06 Extended right hemicolectomy<br>H07 Right hemicolectomy<br>H08 Transverse colectomy<br>H09 Left hemicolectomy<br>H10 Sigmoid colectomy<br>H11 Colectomy NEC*<br>H12 Excision of lesion of colon<br>H13 Bypass of colon<br>H141-142, H144 Caecostomy<br>H151-153 Colostomy<br>H158-159 Exteriorisation of colon<br>H16 Caecotomy/colotomy<br>H17 Open reduction of colonic intussusception/volvulus/obstruction NEC*<br>H33 APER/anterior resection/excision	Emergency mode of admission, primary procedure only

	of rectum<br>G713-715 Bypass of ileum by anastomosis of ileum to colon<br>G721-725 Anastomosis of ileum to colon/rectum/anus<br>G734 Resection of ileo-colic anastomosis	
Emergency urological surgery (UROL)	M021 Nephrectomy and excision of perirenal tissue<br>M022 Nephroureterectomy NEC<br>M023 Bilateral nephrectomy<br>M024 Excision of half of horseshoe kidney<br>M025 Nephrectomy NEC<br>M028 Other specified total excision of kidney<br>M029 Unspecified total excision of kidney<br>M03 Partial excision of kidney<br>M04 Deroofing of cyst/destruction of kidney lesion<br>M051 Open pyeloplasty<br>M052 Open revision of pyeloplasty<br>M18 Excision of ureter<br>M34 Cystectomy<br>M35 Partial excision of bladder<br>M61 Prostatectomy<br>M058 Other specified open repair of kidney<br>M059 Unspecified open repair of kidney<br>M06 Open removal of renal calculus/drainage of kidney<br>M053 Nephropexy<br>M09 Endoscopic fragmentation/extraction of calculus of kidney<br>M10 Endoscopic destruction of lesion of kidney/pyeloplasty/deroofing of cyst<br>M13 Percutaneous biopsy/drainage/aspiration/injection of substance into kidney<br>M14 Extracorporeal fragmentation of calculus of kidney<br>M15 Nephrostomography/other specified or unspecified operations on kidney along nephrostomy tube track<br>M16 Irrigation of kidney/percutaneous	Emergency mode of admission, primary procedure only

	nephrolithotomy NEC/removal of nephrostomy tube/other specified or unspecified operations on kidney<br>M27 Ureteroscopic fragmentation/extraction of calculus of ureter/insertion of ureteric stent/dilation of ureter<br>M28 Endoscopic fragmentation/removal of calculus of ureter<br>M29 Endoscopic insertion/removal of prosthesis into ureter (NEC)/extirpation of lesion of ureter/dilation of ureter<br>M21 Anastomosis of ureter to bladder/ureter/reconstruction of ureter<br>M22 Repair of ureter<br>M26 Nephroscopic fragmentation/extraction of calculus or ureter/insertion of prosthesis<br>M33 Percutaneous insertion of stent into ureter<br>M31 Extracorporeal fragmentation of calculus of ureter<br>M37 Cystourethroplasty/repair of bladder/vesicocolic fistula/other specified or unspecified repair of bladder<br>M38 Cystostomy/other specified or unspecified open drainage of bladder<br>M42 Endoscopic destruction of lesion of bladder<br>M44 Endoscopic removal of calculus/blood clot/foreign body from bladder/other specified or unspecified endoscopic operations on bladder	
Emergency open repair of infra-renal abdominal aortic aneurysm (AAA)	L184-189 Emergency replacement of aneurysmal segment of infra-renal abdominal aorta<br>L194-199 Replacement of aneurysmal segment of infra-renal abdominal aorta<br>L231, L236, L238-239 Plastic repair of aorta<br>L254, L258, L259 Operations on aortic aneurysm NEC*<br>L49 Replacement of aneurysmal iliac artery	Elective or emergency operation codes in conjunction with emergency mode of admission
Emergency	L265 Percutaneous transluminal	Elective or

endovascular repair of infra-renal abdominal aortic aneurysm (EVAR)	insertion of stent into aorta<br>L271 Endovascular insertion of stent graft for infra-renal abdominal aortic aneurysm<br>L275 Endovascular insertion of stent graft for aortic aneurysm of bifurcation NEC<br>L276 Endovascular insertion of stent graft for aorto-uniiliac aneurysm<br>L278 Other specified transluminal insertion of stent graft for aneurysmal segment of aorta<br>L279 Unspecified transluminal insertion of stent graft for aneurysmal segment of aorta<br>L281 Endovascular stenting for infra-renal abdominal aortic aneurysm<br>L285 Endovascular stenting for aortic aneurysm of bifurcation NEC<br>L286 Endovascular stenting for aorto-uniiliac aneurysm<br>L289 Unspecified transluminal operations on aneurysmal segment of aorta<br>Any elective or emergency open AAA code in conjunction with (Y022 [other specified other endovascular placement of stent] and Z346 Z347 Z348 Z349 [abdominal aorta, infra-renal abdominal aorta, aorta NEC]), (O203 O204 O205 O208 O209 [endovascular placement of one or more stent grafts] and Y528 Y53 Y76.9 Y78 [other specified approach to organ through other opening, approach to organ under radiological control, unspecified minimal access to other body cavity, arteriotomy approach to organ using radiological guidance]), (L281 L285 L286 L289) or (L761 L762 L765 L768)	emergency operation codes in conjunction with emergency mode of admission
Emergency carotid endarterectomy (CEA)	L294 Endarterectomy of carotid artery and patch repair of carotid artery<br>L295 Endarterectomy of carotid artery NEC<br>L298 Other specified reconstruction of carotid artery<br>L299 Unspecified reconstruction of carotid artery	Emergency mode of admission

Emergency lower extremity arterial revascularisation (LEAB)	L16 Axillo-femoral bypass/other extra-anatomic aortic bypass<br>L206 Emergency aorto-iliac bypass<br>L216 Aorto-iliac bypass<br>L50 Emergency iliac bypass<br>L51 Iliac bypass<br>L52 Endarterectomy of iliac artery<br>L652-653 Revision of iliac/femoral artery reconstruction<br>L62 Repair/embolectomy of femoral artery<br>L58 Emergency femoro-distal bypass<br>L59 Femoro-distal bypass<br>L60 Endarterectomy/profundoplasty of femoral artery	Elective or emergency operation codes in conjunction with emergency mode of admission
Emergency lower extremity amputation (AMP)	X093 Amputation of leg above knee<br>X094 Amputation of leg through knee<br>X095 Amputation of leg below knee<br>X098 Other specified amputation of leg<br>X099 Unspecified amputation of leg<br>X10 Amputation of foot<br>X11 Amputation of toe<br>X12 Revision of amputation	Emergency mode of admission

OPCS-4 codes used to define emergency medical conditions and emergency surgical procedures, *NEC = not elsewhere classified, **EC = elsewhere classified

eTable 1

OPCS-4 code	Description	Cases	Deaths	Death Rate
U051	Computed tomography of head	232843	19757	8.5%
U212	Computed tomography	172819	10420	6.0%
E852	Non-invasive ventilation	41657	6968	16.7%
E851	Invasive ventilation	13525	3406	25.2%
U201	Echocardiogram	51385	2560	5.0%
L912	Central venous pressure monitoring	10838	2317	21.4%
M479	Insertion of bladder catheter	22550	1831	8.1%
T462	Drainage of ascites	13168	1716	13.0%
X503	Advanced cardiopulmonary resuscitation	1462	1227	83.9%
T124	Insertion of thoracostomy tube	12169	1187	9.8%
W461	Replacement of head of femur	14056	1085	7.7%
T123	Aspiration of pleural cavity	6447	890	13.8%
G459	Upper gastrointestinal endoscopy	14670	843	5.7%
W242	Open reduction/internal fixation of long bone	14155	734	5.2%
W241	Repair of neck of femur fracture	9953	659	6.6%
U071	Computed tomography of chest	9636	638	6.6%
W471	Repair of neck of femur fracture	6396	628	9.8%
X403	Haemodialysis	15077	613	4.1%
U081	Computed tomography of abdomen	10311	569	5.5%
G451	Upper gastrointestinal endoscopy	16609	530	3.2%
**ICD-10 code**	**Description**	**Cases**	**Deaths**	**Death Rate**
J181	Pneumonia	87304	14840	17.0%
J189	Pneumonia	50526	11053	21.9%
I500	Heart failure	37542	5887	15.7%
N390	Urinary tract infection	117054	5640	4.8%
N179	Acute renal failure	26806	5227	19.5%
J180	Pneumonia	8662	5116	59.1%
A419	Sepsis (unspecified)	16378	5111	31.2%
I639	Cerebral infarction	33311	4687	14.1%
J690	Pneumonitis	10259	4047	39.4%
J22X	Acute lower respiratory tract infection	69372	3826	5.5%
J440	Chronic obstructive pulmonary disease	55367	3611	6.5%
S720	Fracture of femur	44206	3567	8.1%
C349	Lung cancer	13593	3548	26.1%
I219	Acute myocardial infarction	28727	3095	10.8%
R54X	Senility	40826	2452	6.0%
R55X	Syncope and collapse	71078	2409	3.4%
I64X	Stroke	12883	2257	17.5%
R060	Abnormalities of breathing	35955	2184	6.1%
I619	Intracerebral haemorrhage	5788	2157	37.3%
K529	Gastroenteritis	60173	2051	3.4%
**HRG code**	**Description**	**Cases**	**Deaths**	**Death**

				Rate
D99	Complex elderly with a respiratory system primary diagnosis	10344	3180	30.7%
DZ1	Lung abscess/empyema/pneumonia	32843	1808	5.5%
AA2	Intracranial procedures except trauma	29972	1237	4.1%
D13	Lobar, atypical or viral pneumonia with cc	6226	1234	19.8%
DZ2	Pulmonary oedema	12727	1066	8.4%
E99	Complex elderly with a cardiac primary diagnosis	5679	992	17.5%
D25	Respiratory neoplasms	4693	924	19.7%
FZ3	Upper gastrointestinal bleed/procedures	83517	913	1.1%
EB0	Cardiac disorders/syncope/heart failure	53349	820	1.5%
A19	Haemorrhagic cerebrovascular disorders	2697	797	29.6%
A99	Complex elderly with a nervous system primary diagnosis	4504	794	17.6%
H99	Complex elderly with a musculoskeletal system primary diagnosis	8638	786	9.1%
S99	Complex elderly with a haematology/infectious disease/poisoning/non-specific primary diagnosis	4942	697	14.1%
L99	Complex elderly with a urinary tract/male reproductive system primary diagnosis	3681	644	17.5%
A22	Non-transient stroke or cerebrovascular accident age > 69 or w cc	5687	627	11.0%
F46	General abdominal disorders age > 69 or w cc	21137	580	2.7%
F36	Large intestinal disorders age > 69 or w cc	12493	460	3.7%
LA0	Kidney transplant/infections	168598	442	0.3%
E28	Cardiac arrest	506	431	85.2%
QZ1	Vascular surgical procedures	21490	417	1.9%

eTable 2

	*Procedure-specific risk-adjusted in-hospital mortality rate by quintile of aggregated risk-adjusted in-hospital mortality rate for other nineteen groups**					
Emergency Condition	*Quintile 1*	*Quintile 2*	*Quintile 3*	*Quintile 4*	*Quintile 5*	*p***
**AMI**	10.7 (0.14)	11.4 (0.14)	11.9 (0.14)	12.7 (0.15)	13.4 (0.14)	p<0.001
**CCF**	15.0 (0.20)	16.2 (0.20)	17.0 (0.21)	17.8 (0.21)	19.8 (0.22)	p<0.001
**CVA**	22.5 (0.20)	23.8 (0.20)	24.9 (0.21)	25.5 (0.21)	28.5 (0.23)	p<0.001
**LRTI**	23.2 (0.17)	25.6 (0.16)	27.2 (0.17)	27.5 (0.17)	29.9 (0.19)	p<0.001
**PE**	7.1 (0.22)	7.6 (0.22)	8.0 (0.24)	8.8 (0.24)	9.5 (0.27)	p<0.001
**UTI**	4.5 (0.08)	5.1 (0.08)	5.4 (0.08)	5.4 (0.08)	5.8 (0.09)	p<0.001
**SEPSIS**	24.3 (0.33)	25.9 (0.35)	29.3 (0.36)	29.3 (0.38)	32.4 (0.39)	p<0.001
**ARREST**	68.6 (1.52)	67.6 (1.60)	68.0 (1.65)	69.2 (1.83)	70.7 (1.66)	p=0.040
**PANC**	5.1 (0.23)	5.2 (0.21)	5.8 (0.22)	6.0 (0.23)	6.6 (0.24)	p<0.001
**NOF**	7.9 (0.15)	9.8 (0.16)	10.2 (0.17)	10.5 (0.17)	11.3 (0.17)	p<0.001
**HERNIA**	2.8 (0.18)	2.9 (0.18)	3.3 (0.20)	3.0 (0.19)	3.8 (0.21)	p<0.001
**APPEND**	0.12 (0.02)	0.21 (0.03)	0.17 (0.03)	0.22 (0.03)	0.25 (0.03)	p=0.002
**PEPTIC**	16.3 (0.93)	16.9 (0.91)	16.1 (0.91)	16.1 (0.90)	18.5 (0.90)	p=0.095
**COLOLAP**	14.0 (0.40)	15.2 (0.43)	16.0 (0.45)	16.3 (0.44)	17.6 (0.45)	p<0.001
**UROL**	3.2 (0.15)	3.6 (0.19)	3.2 (0.18)	3.9 (0.19)	4.5 (0.24)	p<0.001
**AAA**	28.2 (1.29)	32.1 (1.55)	32.9 (1.66)	36.5 (1.87)	38.7 (1.64)	p<0.001
**EVAR**	11.9 (1.96)	12.0 (2.91)	13.6 (3.40)	16.8 (4.20)	17.1 (5.70)	p=0.182
**CEA**	0.26 (0.18)	0.0 (0.0)	0.48 (0.48)	1.9 (0.95)	1.1 (0.80)	p=0.039
**LEAB**	10.7 (0.63)	12.3 (0.81)	11.2 (0.74)	14.5 (0.94)	13.3 (0.83)	p=0.006
**AMP**	8.9 (0.49)	9.7 (0.57)	10.8 (0.58)	11.6 (0.63)	12.1 (0.60)	p<0.001

* Mean % (Standard Error); Quintile 1 = lowest aggregate in-hospital mortality rate, quintile 5 = highest aggregate in-hospital mortality rate.
** Significance of aggregate in-hospital mortality quintile as a predictor of procedure-specific risk-adjusted in-hospital mortality (tested using logistic regression).

eTable 3

	*Procedure-specific risk-adjusted 1-year mortality rate by quintile of aggregated risk-adjusted 1-year mortality rate for other nineteen groups**					
Emergency Condition	*Quintile 1*	*Quintile 2*	*Quintile 3*	*Quintile 4*	*Quintile 5*	*p***
**AMI**	21.4 (0.20)	21.7 (0.18)	22.5 (0.20)	24.3 (0.20)	24.4 (0.20)	p<0.001
**CCF**	40.1 (0.32)	40.5 (0.32)	42.2 (0.32)	43.1 (0.32)	44.6 (0.33)	p<0.001
**CVA**	35.2 (0.25)	36.3 (0.25)	37.8 (0.25)	39.3 (0.26)	40.8 (0.27)	p<0.001
**LRTI**	40.9 (0.22)	41.4 (0.21)	43.4 (0.22)	45.0 (0.23)	46.5 (0.23)	p<0.001
**PE**	20.7 (0.37)	21.1 (0.37)	21.7 (0.37)	22.5 (0.41)	24.5 (0.42)	p<0.001
**UTI**	23.1 (0.18)	24.0 (0.17)	24.3 (0.18)	25.4 (0.18)	26.1 (0.18)	p<0.001
**SEPSIS**	41.2 (0.42)	43.7 (0.45)	45.6 (0.45)	46.7 (0.47)	48.8 (0.48)	p<0.001
**ARREST**	73.1 (1.60)	72.1 (1.65)	76.0 (1.63)	75.8 (1.81)	73.3 (1.85)	p=0.093
**PANC**	9.3 (0.29)	10.0 (0.30)	10.2 (0.30)	10.8 (0.30)	11.5 (0.31)	p<0.001
**NOF**	27.1 (0.28)	27.2 (0.27)	28.8 (0.28)	29.7 (0.28)	30.7 (0.29)	p<0.001
**HERNIA**	8.4 (0.32)	8.4 (0.32)	8.7 (0.32)	9.1 (0.32)	9.3 (0.34)	p=0.08
**APPEND**	0.39 (0.04)	0.44 (0.04)	0.49 (0.04)	0.49 (0.04)	0.57 (0.05)	p=0.001
**PEPTIC**	21.9 (1.06)	21.0 (1.08)	21.8 (1.02)	23.3 (1.04)	23.4 (1.05)	p=0.056
**COLOLAP**	28.5 (0.58)	28.2 (0.60)	29.6 (0.58)	30.4 (0.60)	32.1 (0.62)	p<0.001
**UROL**	19.2 (0.39)	20.0 (0.41)	19.6 (0.43)	20.7 (0.47)	23.7 (0.54)	p<0.001
**AAA**	36.3 (1.60)	34.9 (1.50)	36.3 (1.74)	41.6 (1.87)	44.0 (1.84)	p<0.001
**EVAR**	22.5 (2.75)	26.2 (3.47)	12.3 (3.30)	20.2 (6.73)	36.0 (9.29)	p=0.954
**CEA**	5.3 (1.03)	7.0 (1.66)	6.4 (1.64)	11.0 (2.95)	8.2 (2.58)	p=0.169
**LEAB**	24.7 (0.99)	24.4 (1.02)	27.2 (1.24)	28.7 (1.25)	30.3 (1.34)	p<0.001
**AMP**	24.9 (0.84)	24.3 (0.85)	26.6 (0.93)	26.5 (0.88)	28.1 (0.97)	p<0.001

* Mean % (Standard Error); Quintile 1 = lowest aggregate 1-year mortality rate, quintile 5 = highest aggregate 1-year mortality rate.
** Significance of aggregate 1-year mortality quintile as a predictor of procedure-specific risk-adjusted 1-year mortality (tested using logistic regression).

eTable 4

	*Procedure-specific risk-adjusted 28-day emergency readmission rate by quintile of aggregated risk-adjusted 28-day emergency readmission rate for other nineteen groups**					
Emergency Condition	*Quintile 1*	*Quintile 2*	*Quintile 3*	*Quintile 4*	*Quintile 5*	*p***
**AMI**	14.2 (0.15)	15.2 (0.18)	15.5 (0.18)	15.6 (0.17)	16.6 (0.18)	p<0.001
**CCF**	16.7 (0.22)	18.6 (0.24)	19.2 (0.23)	19.7 (0.25)	20.4 (0.24)	p<0.001
**CVA**	9.0 (0.14)	9.9 (0.15)	10.6 (0.16)	10.7 (0.16)	11.6 (0.16)	p<0.001
**LRTI**	13.5 (0.14)	15.0 (0.15)	15.6 (0.15)	16.0 (0.16)	16.7 (0.15)	p<0.001
**PE**	12.6 (0.30)	12.7 (0.30)	14.0 (0.32)	14.3 (0.32)	15.1 (0.34)	p<0.001
**UTI**	13.9 (0.15)	15.4 (0.15)	16.4 (0.15)	16.8 (0.15)	17.3 (0.14)	p<0.001
**SEPSIS**	18.1 (0.35)	18.0 (0.33)	18.1 (0.33)	19.2 (0.36)	19.6 (0.36)	p<0.001
**ARREST**	12.7 (1.23)	12.1 (1.18)	14.8 (1.29)	14.8 (1.36)	16.7 (1.36)	p=0.005
**PANC**	13.1 (0.35)	14.4 (0.37)	14.4 (0.37)	14.8 (0.37)	14.9 (0.36)	p<0.001
**NOF**	9.9 (0.17)	10.8 (0.17)	11.8 (0.19)	12.1 (0.20)	12.8 (0.20)	p<0.001
**HERNIA**	8.3 (0.33)	9.3 (0.33)	10.5 (0.35)	10.5 (0.36)	10.7 (0.37)	p<0.001
**APPEND**	7.3 (0.17)	8.1 (0.16)	8.3 (0.17)	8.3 (0.18)	8.2 (0.17)	p<0.001
**PEPTIC**	8.5 (0.72)	9.9 (0.77)	10.1 (0.77)	9.6 (0.77)	10.3 (0.77)	p=0.190
**COLOLAP**	13.1 (0.43)	13.9 (0.43)	14.3 (0.45)	15.1 (0.47)	14.9 (0.46)	p<0.001
**UROL**	17.2 (0.46)	17.9 (0.45)	18.3 (0.42)	19.0 (0.39)	18.9 (0.45)	p<0.001
**AAA**	10.6 (1.05)	11.5 (1.14)	10.9 (1.12)	15.0 (1.25)	11.6 (1.26)	p=0.097
**EVAR**	20.1 (5.03)	6.6 (3.83)	18.5 (2.96)	19.8 (3.55)	19.2 (4.53)	p=0.245
**CEA**	9.7 (2.28)	6.5 (1.82)	11.5 (2.44)	7.3 (1.47)	10.0 (1.88)	p=0.967
**LEAB**	16.6 (0.99)	20.3 (1.18)	18.3 (0.92)	20.0 (1.01)	21.7 (1.14)	p<0.001
**AMP**	13.0 (0.66)	13.4 (0.69)	16.6 (0.72)	15.5 (0.74)	16.7 (0.79)	p<0.001

* Mean % (Standard Error); Quintile 1 = lowest aggregate 28-day emergency readmission rate, quintile 5 = highest aggregate 28-day emergency readmission rate.
** Significance of aggregate 28-day emergency readmission quintile as a predictor of procedure-specific risk-adjusted 28-day emergency readmission (tested using logistic regression).

eTable 5

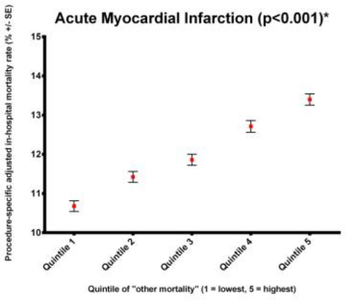
Acute Myocardial Infarction (p<0.001)*
Procedure-specific adjusted in-hospital mortality rate (% +/- SE)
Quintile 1
Quintile 2
Quintile 3
Quintile 4
Quintile 5
Quintile of "other mortality" (1 = lowest, 5 = highest)

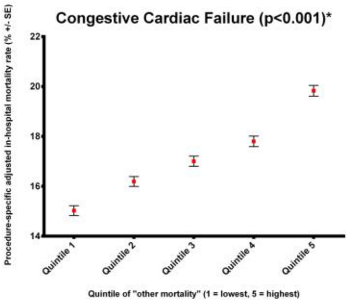
Congestive Cardiac Failure (p<0.001)*
Procedure-specific adjusted in-hospital mortality rate (% +/- SE)
Quintile 1
Quintile 2
Quintile 3
Quintile 4
Quintile 5
Quintile of "other mortality" (1 = lowest, 5 = highest)

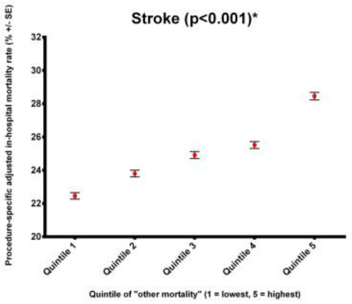
Stroke (p<0.001)*
Procedure-specific adjusted in-hospital mortality rate (% +/- SE)
Quintile 1
Quintile 2
Quintile 3
Quintile 4
Quintile 5
Quintile of "other mortality" (1 = lowest, 5 = highest)

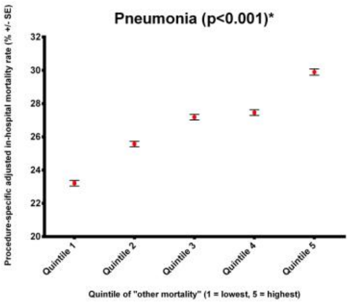
Pneumonia (p<0.001)*
Procedure-specific adjusted in-hospital mortality rate (% +/- SE)
Quintile 1
Quintile 2
Quintile 3
Quintile 4
Quintile 5
Quintile of "other mortality" (1 = lowest, 5 = highest)

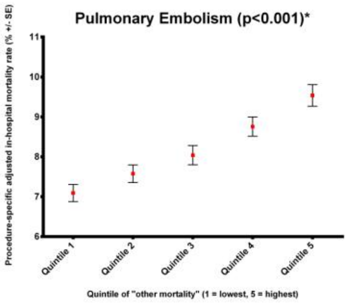
Pulmonary Embolism (p<0.001)*
Procedure-specific adjusted in-hospital mortality rate (% +/- SE)
Quintile 1
Quintile 2
Quintile 3
Quintile 4
Quintile 5
Quintile of "other mortality" (1 = lowest, 5 = highest)

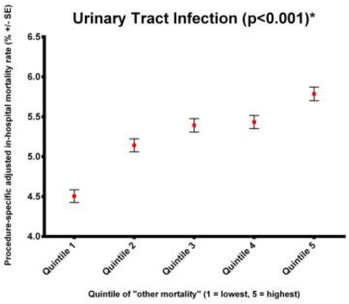
Urinary Tract Infection (p<0.001)*
Procedure-specific adjusted in-hospital mortality rate (% +/- SE)
Quintile 1
Quintile 2
Quintile 3
Quintile 4
Quintile 5
Quintile of "other mortality" (1 = lowest, 5 = highest)

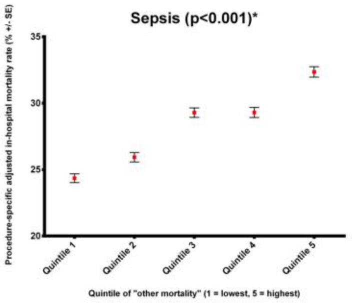
Sepsis (p<0.001)*
Procedure-specific adjusted in-hospital mortality rate (% +/- SE)
Quintile 1
Quintile 2
Quintile 3
Quintile 4
Quintile 5
Quintile of "other mortality" (1 = lowest, 5 = highest)

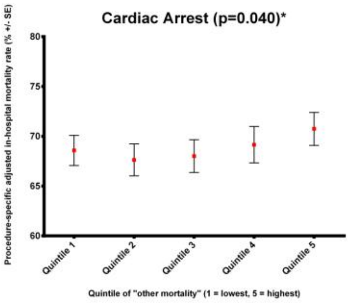
Cardiac Arrest (p=0.040)*
Procedure-specific adjusted in-hospital mortality rate (% +/- SE)
Quintile 1
Quintile 2
Quintile 3
Quintile 4
Quintile 5
Quintile of "other mortality" (1 = lowest, 5 = highest)

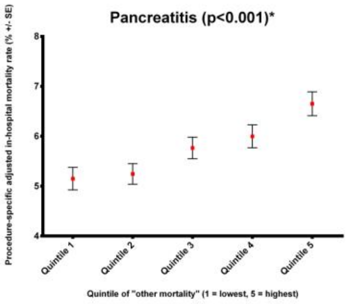
Pancreatitis (p<0.001)*
Procedure-specific adjusted in-hospital mortality rate (% +/- SE)
Quintile 1
Quintile 2
Quintile 3
Quintile 4
Quintile 5
Quintile of "other mortality" (1 = lowest, 5 = highest)

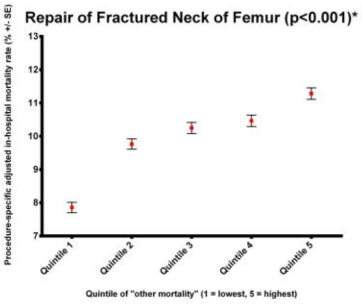
Repair of Fractured Neck of Femur (p<0.001)*
Procedure-specific adjusted in-hospital mortality rate (% +/- SE)
Quintile 1
Quintile 2
Quintile 3
Quintile 4
Quintile 5
Quintile of "other mortality" (1 = lowest, 5 = highest)

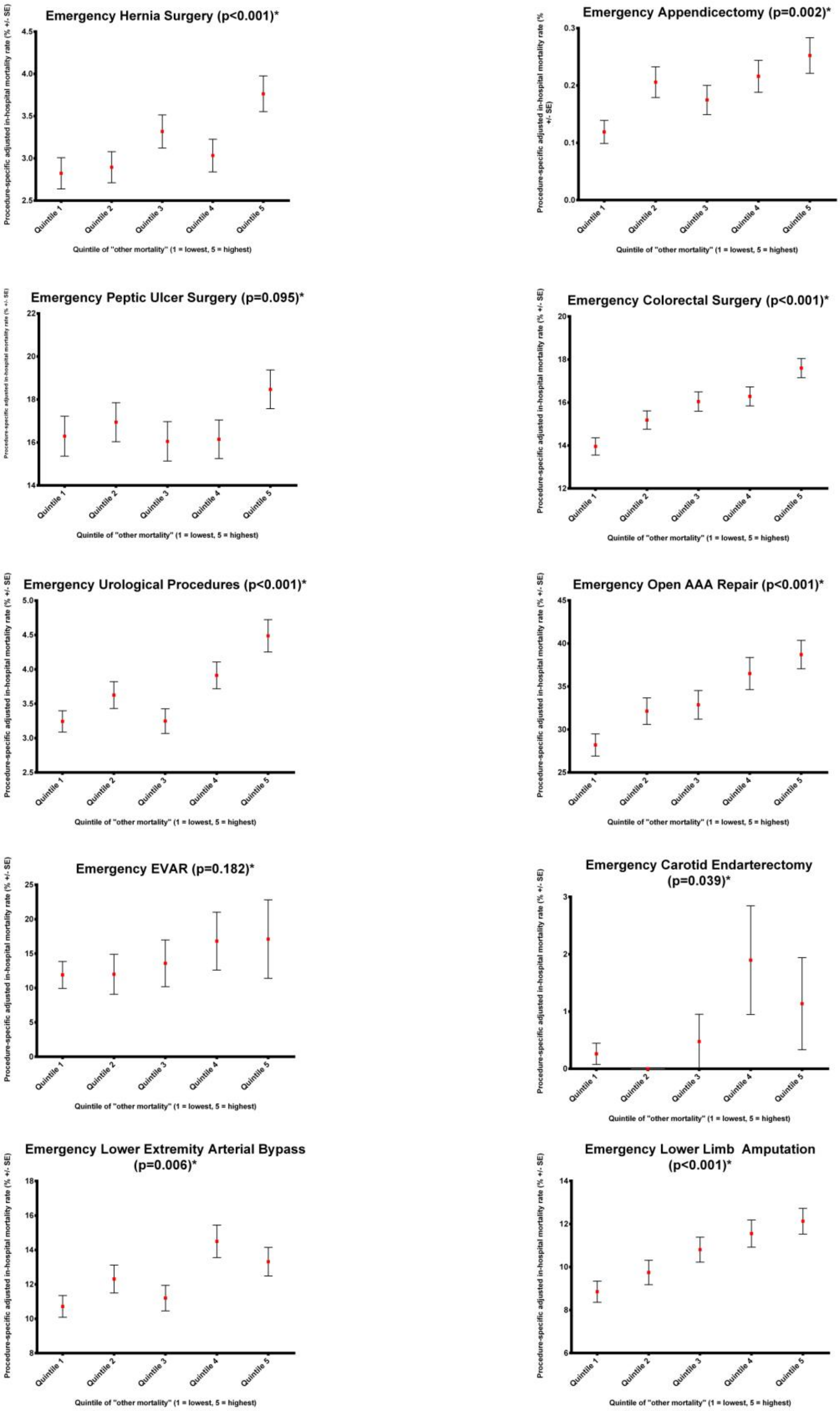
Emergency Hernia Surgery (p<0.001)*
Emergency Appendicectomy (p=0.002)*
Emergency Peptic Ulcer Surgery (p=0.095)*
Emergency Colorectal Surgery (p<0.001)*
Emergency Urological Procedures (p<0.001)*
Emergency Open AAA Repair (p<0.001)*
Emergency EVAR (p=0.182)*
Emergency Carotid Endarterectomy (p=0.039)*
Emergency Lower Extremity Arterial Bypass (p=0.006)*
Emergency Lower Limb Amputation (p<0.001)*
Procedure-specific adjusted in-hospital mortality rate (% +/- SE)
Quintile 1
Quintile 2
Quintile 3
Quintile 4
Quintile 5
Quintile of "other mortality" (1 = lowest, 5 = highest)

eFigure 1

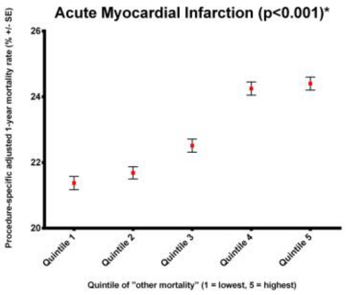
Acute Myocardial Infarction (p<0.001)*
Procedure-specific adjusted 1-year mortality rate (% +/- SE)
26
24
22
20
Quintile 1
Quintile 2
Quintile 3
Quintile 4
Quintile 5
Quintile of "other mortality" (1 = lowest, 5 = highest)

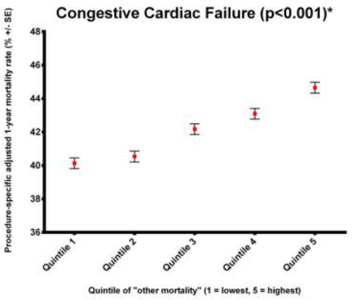
Congestive Cardiac Failure (p<0.001)*
Procedure-specific adjusted 1-year mortality rate (% +/- SE)
48
46
44
42
40
38
36
Quintile 1
Quintile 2
Quintile 3
Quintile 4
Quintile 5
Quintile of "other mortality" (1 = lowest, 5 = highest)

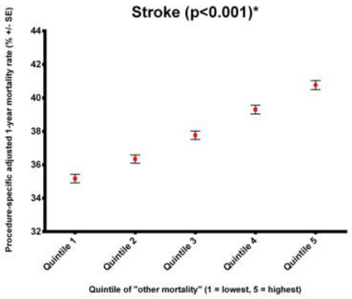
Stroke (p<0.001)*
Procedure-specific adjusted 1-year mortality rate (% +/- SE)
44
42
40
38
36
34
32
Quintile 1
Quintile 2
Quintile 3
Quintile 4
Quintile 5
Quintile of "other mortality" (1 = lowest, 5 = highest)

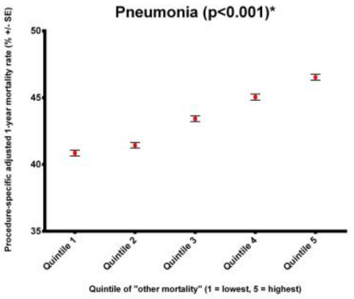
Pneumonia (p<0.001)*
Procedure-specific adjusted 1-year mortality rate (% +/- SE)
50
45
40
35
Quintile 1
Quintile 2
Quintile 3
Quintile 4
Quintile 5
Quintile of "other mortality" (1 = lowest, 5 = highest)

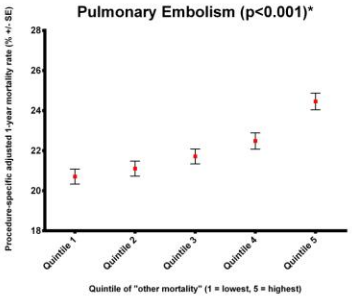
Pulmonary Embolism (p<0.001)*
Procedure-specific adjusted 1-year mortality rate (% +/- SE)
28
26
24
22
20
18
Quintile 1
Quintile 2
Quintile 3
Quintile 4
Quintile 5
Quintile of "other mortality" (1 = lowest, 5 = highest)

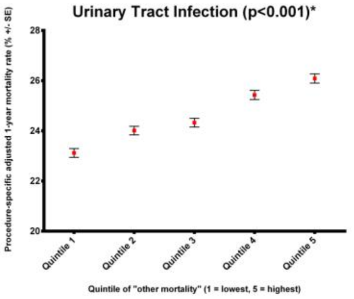
Urinary Tract Infection (p<0.001)*
Procedure-specific adjusted 1-year mortality rate (% +/- SE)
28
26
24
22
20
Quintile 1
Quintile 2
Quintile 3
Quintile 4
Quintile 5
Quintile of "other mortality" (1 = lowest, 5 = highest)

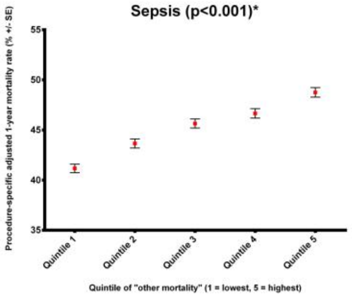
Sepsis (p<0.001)*
Procedure-specific adjusted 1-year mortality rate (% +/- SE)
55
50
45
40
35
Quintile 1
Quintile 2
Quintile 3
Quintile 4
Quintile 5
Quintile of "other mortality" (1 = lowest, 5 = highest)

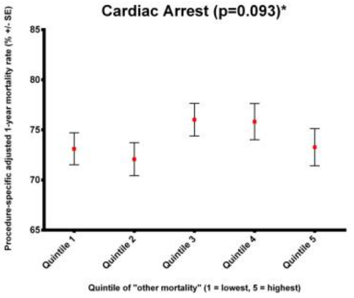
Cardiac Arrest (p=0.093)*
Procedure-specific adjusted 1-year mortality rate (% +/- SE)
85
80
75
70
65
Quintile 1
Quintile 2
Quintile 3
Quintile 4
Quintile 5
Quintile of "other mortality" (1 = lowest, 5 = highest)

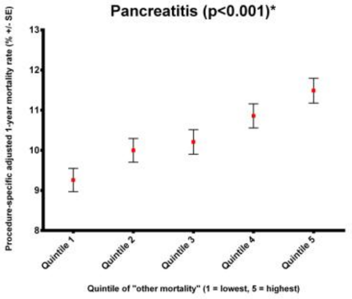
Pancreatitis (p<0.001)*
Procedure-specific adjusted 1-year mortality rate (% +/- SE)
13
12
11
10
9
8
Quintile 1
Quintile 2
Quintile 3
Quintile 4
Quintile 5
Quintile of "other mortality" (1 = lowest, 5 = highest)

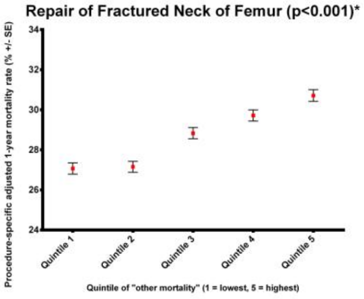
Repair of Fractured Neck of Femur (p<0.001)*
Procedure-specific adjusted 1-year mortality rate (% +/- SE)
34
32
30
28
26
24
Quintile 1
Quintile 2
Quintile 3
Quintile 4
Quintile 5
Quintile of "other mortality" (1 = lowest, 5 = highest)

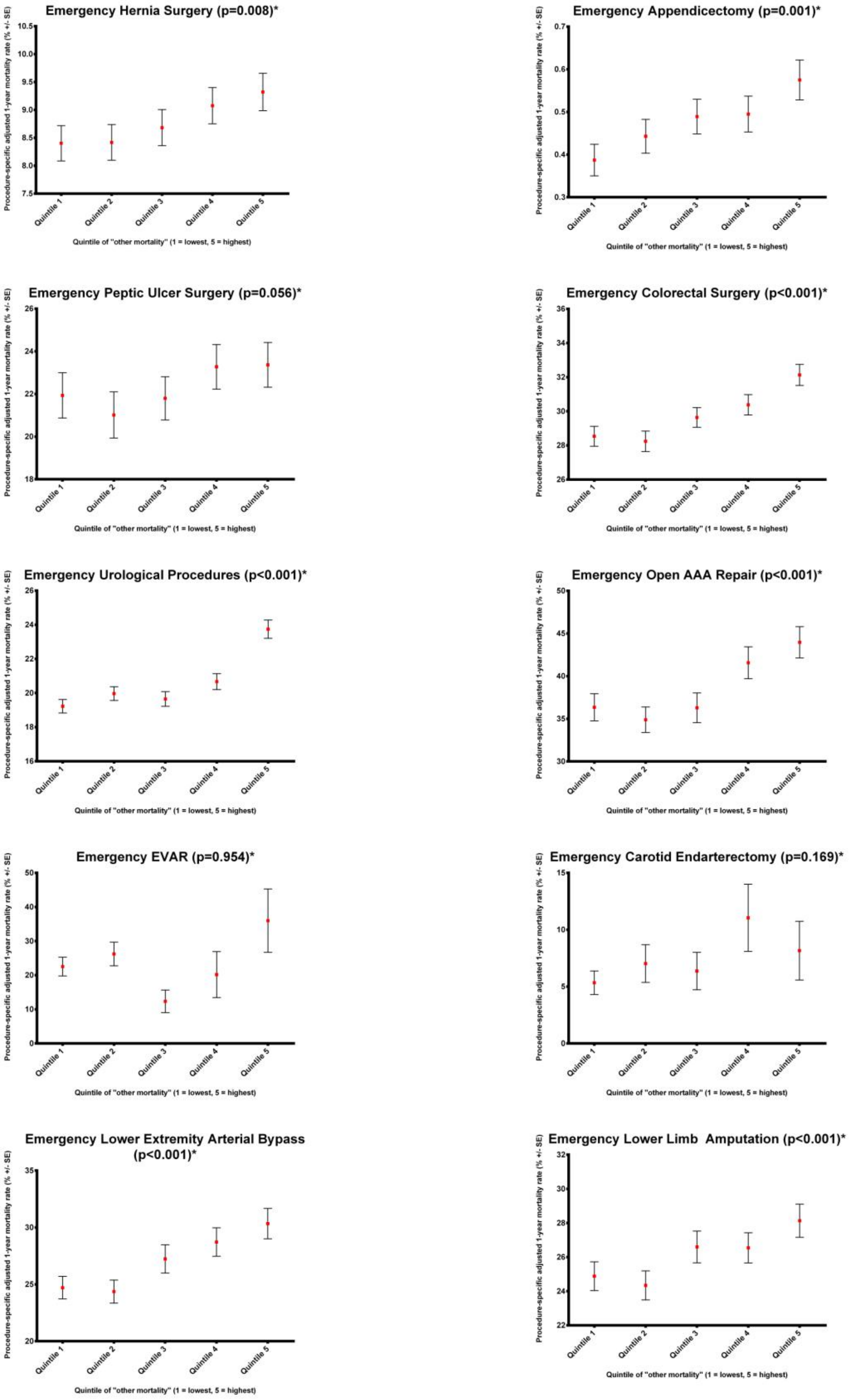
Emergency Hernia Surgery (p=0.008)*
Emergency Appendicectomy (p=0.001)*
Emergency Peptic Ulcer Surgery (p=0.056)*
Emergency Colorectal Surgery (p<0.001)*
Emergency Urological Procedures (p<0.001)*
Emergency Open AAA Repair (p<0.001)*
Emergency EVAR (p=0.954)*
Emergency Carotid Endarterectomy (p=0.169)*
Emergency Lower Extremity Arterial Bypass (p<0.001)*
Emergency Lower Limb Amputation (p<0.001)*
Procedure-specific adjusted 1-year mortality rate (% +/- SE)
Quintile 1
Quintile 2
Quintile 3
Quintile 4
Quintile 5
Quintile of "other mortality" (1 = lowest, 5 = highest)

eFigure 2

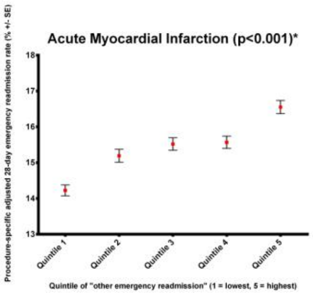
Acute Myocardial Infarction (p<0.001)*
Procedure-specific adjusted 28-day emergency readmission rate (% +/- SE)
13
14
15
16
17
18
Quintile 1
Quintile 2
Quintile 3
Quintile 4
Quintile 5
Quintile of "other emergency readmission" (1 = lowest, 5 = highest)

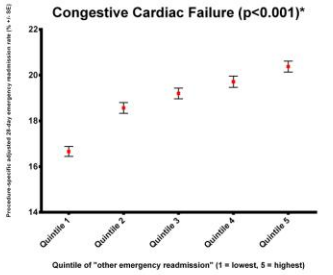
Congestive Cardiac Failure (p<0.001)*
Procedure-specific adjusted 28-day emergency readmission rate (% +/- SE)
14
16
18
20
22
Quintile 1
Quintile 2
Quintile 3
Quintile 4
Quintile 5
Quintile of "other emergency readmission" (1 = lowest, 5 = highest)

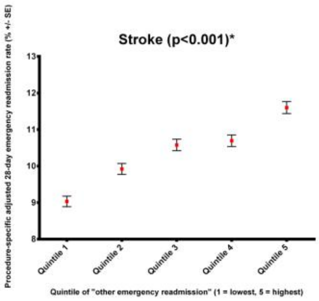
Stroke (p<0.001)*
Procedure-specific adjusted 28-day emergency readmission rate (% +/- SE)
8
9
10
11
12
13
Quintile 1
Quintile 2
Quintile 3
Quintile 4
Quintile 5
Quintile of "other emergency readmission" (1 = lowest, 5 = highest)

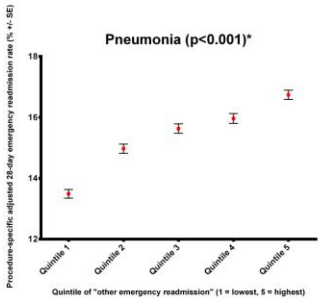
Pneumonia (p<0.001)*
Procedure-specific adjusted 28-day emergency readmission rate (% +/- SE)
12
14
16
18
Quintile 1
Quintile 2
Quintile 3
Quintile 4
Quintile 5
Quintile of "other emergency readmission" (1 = lowest, 5 = highest)

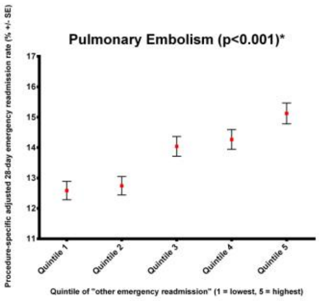
Pulmonary Embolism (p<0.001)*
Procedure-specific adjusted 28-day emergency readmission rate (% +/- SE)
11
12
13
14
15
16
17
Quintile 1
Quintile 2
Quintile 3
Quintile 4
Quintile 5
Quintile of "other emergency readmission" (1 = lowest, 5 = highest)

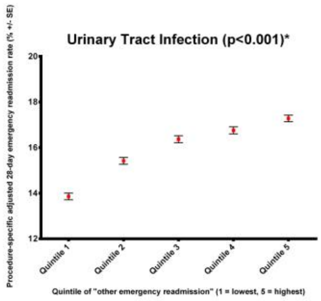
Urinary Tract Infection (p<0.001)*
Procedure-specific adjusted 28-day emergency readmission rate (% +/- SE)
12
14
16
18
20
Quintile 1
Quintile 2
Quintile 3
Quintile 4
Quintile 5
Quintile of "other emergency readmission" (1 = lowest, 5 = highest)

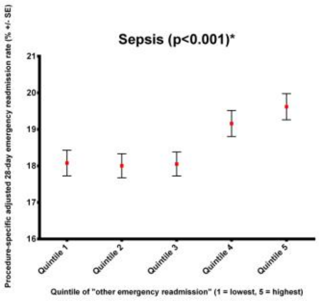
Sepsis (p<0.001)*
Procedure-specific adjusted 28-day emergency readmission rate (% +/- SE)
16
17
18
19
20
21
Quintile 1
Quintile 2
Quintile 3
Quintile 4
Quintile 5
Quintile of "other emergency readmission" (1 = lowest, 5 = highest)

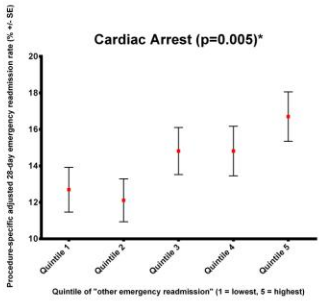
Cardiac Arrest (p=0.005)*
Procedure-specific adjusted 28-day emergency readmission rate (% +/- SE)
10
12
14
16
18
20
Quintile 1
Quintile 2
Quintile 3
Quintile 4
Quintile 5
Quintile of "other emergency readmission" (1 = lowest, 5 = highest)

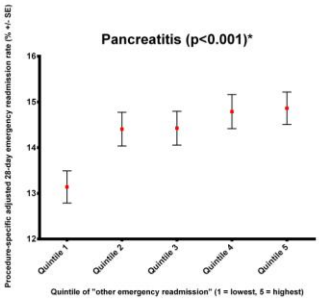
Pancreatitis (p<0.001)*
Procedure-specific adjusted 28-day emergency readmission rate (% +/- SE)
12
13
14
15
16
Quintile 1
Quintile 2
Quintile 3
Quintile 4
Quintile 5
Quintile of "other emergency readmission" (1 = lowest, 5 = highest)

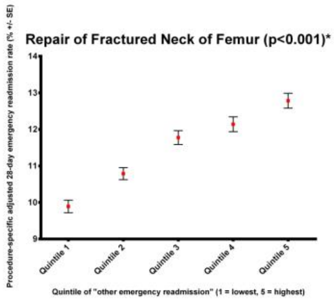
Repair of Fractured Neck of Femur (p<0.001)*
Procedure-specific adjusted 28-day emergency readmission rate (% +/- SE)
9
10
11
12
13
14
Quintile 1
Quintile 2
Quintile 3
Quintile 4
Quintile 5
Quintile of "other emergency readmission" (1 = lowest, 5 = highest)

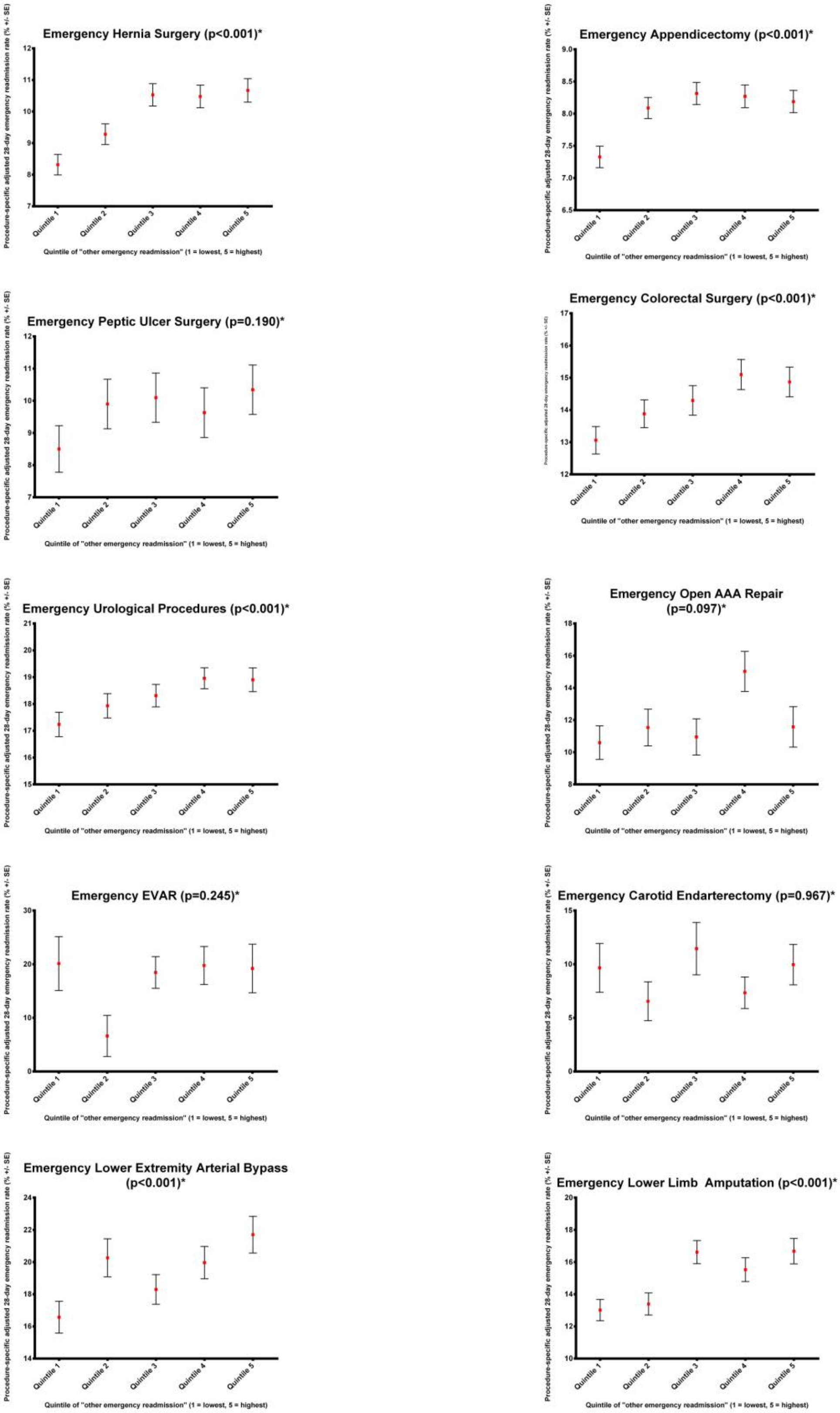
Emergency Hernia Surgery (p<0.001)*
Emergency Appendicectomy (p<0.001)*
Emergency Peptic Ulcer Surgery (p=0.190)*
Emergency Colorectal Surgery (p<0.001)*
Emergency Urological Procedures (p<0.001)*
Emergency Open AAA Repair (p=0.097)*
Emergency EVAR (p=0.245)*
Emergency Carotid Endarterectomy (p=0.967)*
Emergency Lower Extremity Arterial Bypass (p<0.001)*
Emergency Lower Limb Amputation (p<0.001)*
Procedure-specific adjusted 28-day emergency readmission rate (% +/- SE)
Quintile 1
Quintile 2
Quintile 3
Quintile 4
Quintile 5
Quintile of "other emergency readmission" (1 = lowest, 5 = highest)

eFigure 3
